# Immunosuppressive tumor microenvironment in extraskeletal myxoid chondrosarcoma: A case of pleural metastases

**DOI:** 10.1111/1759-7714.14613

**Published:** 2022-08-16

**Authors:** Ryosuke Ogata, Hiroshi Soda, Hiroaki Senju, Masaki Fujioka, Midori Shimada, Koki Yamashita, Satoshi Irifune, Ryuta Tagawa, Yosuke Dotsu, Keisuke Iwasaki, Hirokazu Taniguchi, Shinnosuke Takemoto, Yuichi Fukuda, Hiroshi Mukae

**Affiliations:** ^1^ Department of Respiratory Medicine Sasebo City General Hospital Nagasaki Japan; ^2^ Department of Respiratory Medicine Senju Hospital Nagasaki Japan; ^3^ Department of Plastic and Reconstructive Surgery National Hospital Organization Nagasaki Medical Center Nagasaki Japan; ^4^ Clinical Research Center Nagasaki University Hospital Nagasaki Japan; ^5^ Department of Respiratory Medicine Nagasaki University Graduate School of Biomedical Sciences Nagasaki Japan; ^6^ Department of Pathology Sasebo City General Hospital Nagasaki Japan

**Keywords:** sarcoma, transforming growth factor‐β1, tumor‐associated macrophages, vascular endothelial growth factor

## Abstract

Extraskeletal myxoid chondrosarcoma (EMCS) is an undifferentiated mesenchymal malignancy; however, its immune microenvironment remains to be elucidated. The case of a 34‐year‐old woman who developed EMCS metastasizing to the pleura is presented here. The pleural EMCS showed hypervascularity, absent PD‐L1 expression, and a lack of tumor mutational burden and pathogenic variants. Immunohistological examination of the pleural lesions showed predominant M2 macrophages and sparse CD8^+^ T cells. EMCS and the tumor stroma were positive for transforming growth factor‐β1 (TGF‐β1) and vascular endothelial growth factor (VEGF). In contrast, a small number of the stromal vessels were positive for hypoxia inducible factor‐1α (HIF‐1α). TGF‐β1 and VEGF in the tumor stroma and low antigenicity of the tumor cells may help explain how EMCS induced the immunosuppressive microenvironment. These findings may encourage investigators to explore novel combined immunotherapy for EMCS, such as TGF‐β1 and VEGF inhibitors, and specific therapy for enhancing tumor antigens.

## Graphical abstract

Extraskeletal myxoid chondrosarcoma cells secrete transforming growth factor‐β1 and vascular endothelial growth factor, which could induce the immunosuppressive microenvironment with a predominance of M2 macrophages, lack of CD8^+^ cytotoxic T cells, and increase of small vessels. Tumor cells have low tumor mutational burden (TMB) and no PD‐L1 expression.

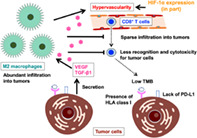

## INTRODUCTION

Extraskeletal myxoid chondrosarcoma (EMCS) is a rare mesenchymal malignancy of uncertain differentiation. Despite the name, there has been no convincing evidence of cartilaginous differentiation. EMCS is considered to be different from chondrosarcoma.^1^ Although anthracycline‐based chemotherapy is the standard therapy for advanced EMCS, this regimen has limited antitumor activity, and novel therapeutic approaches are therefore required.^1^, ^2^ Since immunotherapy has been developed for various malignancies, it is important to examine whether EMCS may be a candidate for immunotherapy.^3^ However, little is known about the underlying immune microenvironment of EMCS.^4^, ^5^ To the best of our knowledge, this is the first report of the combination of immunohistochemical and genomic analyses, using FoundationOne CDx next‐generation sequencing of the immune microenvironment in a case of EMCS.

## CASE REPORT

**FIGURE 1:**
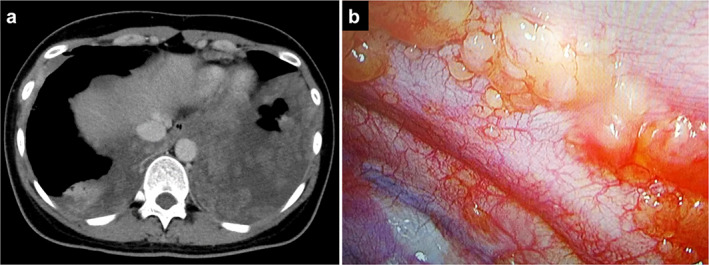
A patient with extraskeletal myxoid chondrosarcoma metastasizing to the pleura. (a) Chest computed tomography shows bilateral pleural effusions with multiple pleural nodules. (b) Thoracoscopic view shows multiple yellowish nodules with hypervascularity on the left parietal pleura

**FIGURE 2:**
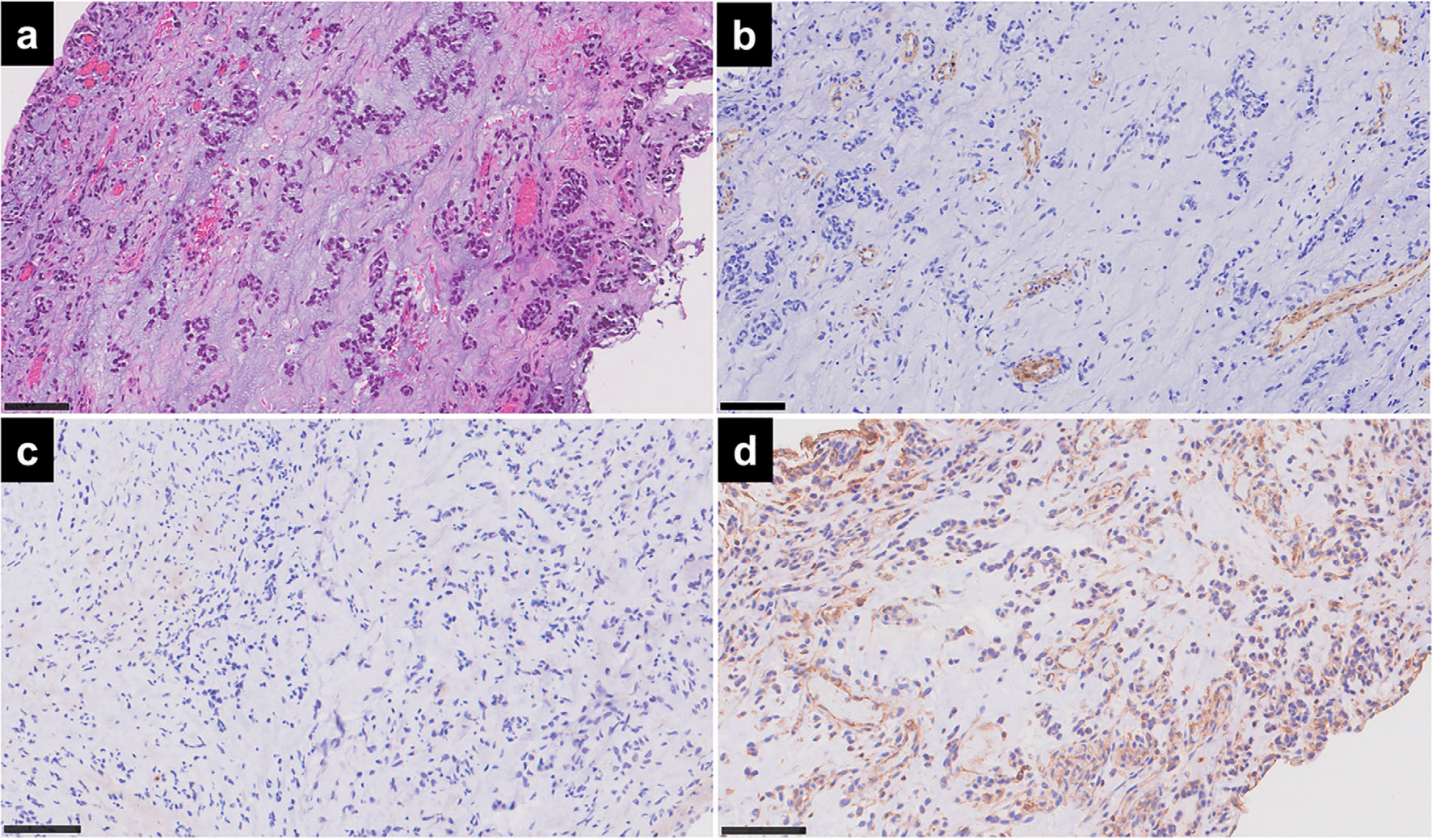
Photomicrographs of the thoracoscopic pleural biopsy specimen taken from a patient with extraskeletal myxoid chondrosarcoma. Scale bars indicate 100 μm. (a) Nests of polygonal tumor cells with abundant myxoid stroma and small vessels (hematoxylin & eosin stain). The immunohistological study shows (b) smooth muscle actin‐positive vessels, (c) the absence of PD‐L1, and (d) the presence of HLA class I

A 34‐year‐old woman who had never smoked was referred to our department with prickling chest pain and bilateral pleural effusions. Three years earlier, she had undergone surgery and radiotherapy for EMCS of the right knee at another hospital. Physical examination and laboratory tests were unremarkable. Chest computed tomography showed bilateral pleural effusions with multiple pleural nodules (Figure 1a). Positron emission tomography showed uptake of ^18^F‐fluorodeoxyglucose into the pleural nodules. Although it was suspected that the nodules were malignant, thoracentesis failed to establish a diagnosis. The patient then underwent thoracoscopic biopsy of the left pleural nodules. Thoracoscopic examination showed yellowish, multinodular lesions with hypervascularity on the parietal pleura (Figure 1b). Pathologically, the biopsy specimens showed nests of polygonal tumor cells with abundant myxoid stroma and small vessels (Figure 2a,b). FoundationOne CDx next‐generation DNA sequencing (Foundation Medicine, Inc.) of the lesions showed only *NR4A3‐EWSR1* fusion, tumor mutational burden of 4 mutations/Mb, and stable microsatellite status. The pathological findings and disease‐specific *NR4A3‐EWSR1* fusion led to the diagnosis of EMCS metastasizing to the pleura. The patient received anthracycline‐based chemotherapy at another hospital, and the disease remained stable.

**FIGURE 3:**
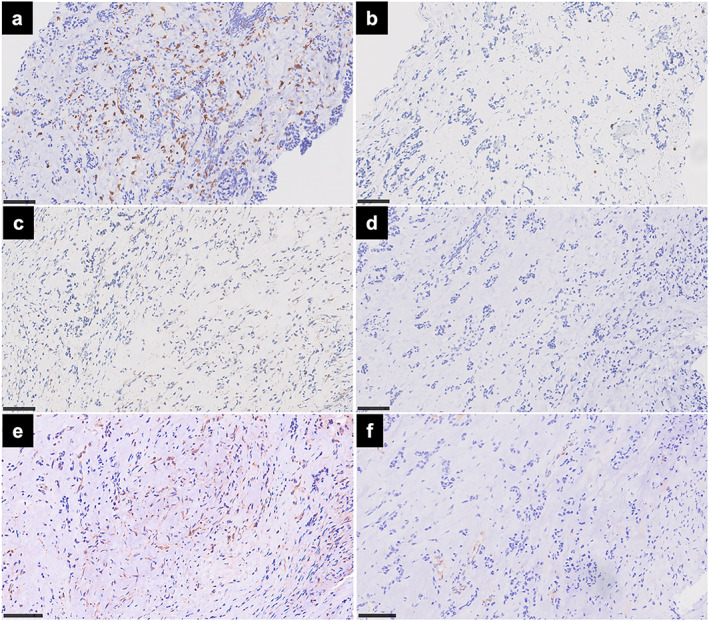
Immunohistological investigation of immune cells in the thoracoscopic pleural biopsy specimen taken from a patient with extraskeletal myxoid chondrosarcoma. Scale bars indicate 100 μm. (a) The predominance of CD163^+^ M2 macrophages. (b) The lack of CD8^+^ cytotoxic T cells, (c) CD4^+^ helper T cells, and (d) FOXP3^+^ regulatory T cells. (e) Vimentin‐positive and (f) smooth muscle actin‐negative fibroblasts

**FIGURE 4:**
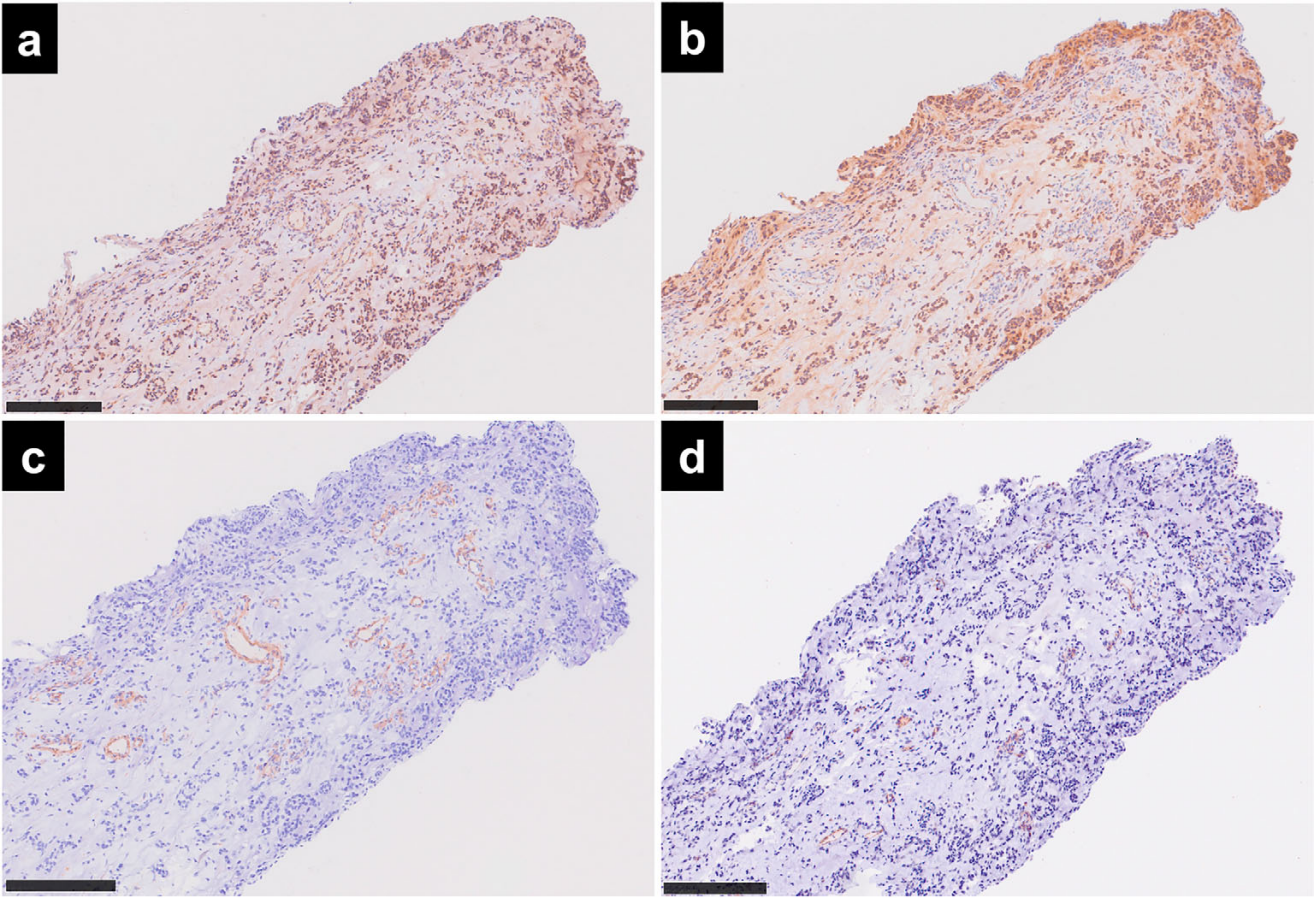
Immunohistological study of tumor‐associated cytokines in the thoracoscopic pleural biopsy specimen taken from a patient with extraskeletal myxoid chondrosarcoma. Scale bars indicate 250 μm. Tumor cells and myxoid stroma positive for (a) transforming growth factor‐β1 and (b) vascular endothelial growth factor. (c) The tumor stromal vessels were positive for smooth muscle actin. (d) A small number of the stromal vessels were positive for hypoxia inducible factor‐1α

On immunohistological examination of the pleural lesions, the EMCS cells did not express PD‐L1 (clone 22–8, Abcam; Figure 2c). Although tumor mutational burden was low, HLA class I was seen in the tumor cells (Figure 2d). In the tumor stroma, CD163^+^ M2 immunosuppressive macrophages were predominant (Figure 3a), whereas few CD8^+^ cytotoxic T cells were recruited (Figure 3b). Neither CD4^+^ helper T cells nor FOXP3^+^ regulatory T cells were observed (Figure 3c,d). Vimentin‐positive fibroblasts were negative for smooth muscle actin (SMA), suggesting that they were not immunosuppressive tumor‐associated fibroblasts (Figure 3e,f). Furthermore, EMCS cells and the tumor stroma were positive for vascular endothelial growth factor (VEGF; Figure 4a). and transforming growth factor‐β1 (TGF‐β1; Figure 4b). Despite a large number of stromal vessels (Figure 4c), a small number of the stromal vessels were positive for hypoxia inducible factor‐1α (HIF‐1α; Figure 4d). The antibody clones used were as follows: CD4 (4B12); CD8 (4B11); CD163 (10D6); FOXP3 (236A/E7); HIF‐1α (54/ HIF‐1α); HLA class I (EMR8‐5); PD‐L1 (28–8); SMA (1A4); TGF‐β1 (R&D, catalog no. AB‐246‐NA); VEGF (Abcam, catalog no. ab46154); and vimentin (V9).

## DISCUSSION

The present study showed the following important findings. The tumor stroma of EMCS mainly contained M2 macrophages, VEGF, and TGF‐β1, whereas few CD8^+^ T cells were recruited. Furthermore, the EMCS cells had low tumor mutational burden and few pathogenic variants.

CD163^+^ M2 macrophages were predominant in the tumor stroma, whereas few CD8^+^ T cells were recruited. Previous cohort studies of EMCS also reported that CD163^+^ M2 macrophages were the most enriched cell type in the stroma, and few CD8^+^ T cells and CD4^+^ T cells were detected.^4^, ^5^ In another investigation of sarcomas, one case of EMCS showed CD8^+^ T cells of <5%, CD4^+^ T cells of <5%, and no FOXP3^+^ T cells. Two cases had no PD‐L1^+^ tumor cells.^6^ However, this pathophysiology was not mentioned in detail.

In the present study, the EMCS cells and tumor stroma were positive for TGF‐β1 and VEGF, suggesting that the EMCS cells secreted TGF‐β1 and VEGF. In a phase II trial of pazopanib for EMCS, transcriptome‐sequencing revealed high expression level of *VEGFA* gene, although *TGFB1* gene was not investigated.^4^ The expression levels of VEGFA and TGF‐β1 protein were not studied in this trial. Both TGF‐β1 and VEGF typically promote the polarization of macrophages to an immunosuppressive M2 phenotype. M2 macrophages themselves potentially secrete TGF‐β1 and VEGF and inhibit the recruitment and cytotoxic function of CD8^+^ T cells.^7^, ^8^ VEGF may be involved in hypervascularity in the tumor stroma, which could prevent CD8^+^ T cells from trafficking into the tumor.^9^ Furthermore, HIF‐1α‐positive stromal vessels may participate partially in hypervascularity.^10^ The crosstalk of TGF‐β1, VEGF, M2 macrophages, and hypervascularity in the stroma possibly produces the immunosuppressive environment of EMCS.

The EMCS cells showed low tumor mutational burden and few pathogenic variants, in addition to less infiltration of CD8^+^ T cells. In a cohort study of EMSC, the next‐generation DNA sequencing test detected only *NR4A3‐EWSR1* fusion and low tumor mutational burden.^11^ This test detected no other pathogenic variants that potentially induced genomic instability, such as mismatch repair genes and homologous recombinant repair genes. Low tumor mutational burden and few pathogenic variants may lead to low tumor antigenicity, causing the decreased recruitment of CD8^+^ T cells into the tumors.^12^

The present study has several limitations. First, the discrepancy of infiltration between M2 macrophages and regulatory T cells was observed. In immune gene signature of hepatocellular carcinoma, M2 macrophages and regulatory T cells were mutually exclusive.^13^ M2 macrophages and regulatory T cells were seen in the “noninflamed” and “inflamed” clusters, respectively. Also, multiplex immunofluorescence analysis of non‐small cell lung cancer showed that M2 macrophages correlated poorly with regulatory T cells.^14^ Further studies are needed to identify the different role of M2 macrophages and regulatory T cells on tumor immune evasion.

The second limitation of the present study is that HIF‐1α expression was seen in a small number of the stromal vessels, but not EMCS cells expressing VEGF. Sp1 transcriptional factor as well as HIF‐1α upregulates the expression of *VEGF* gene.^15^ Several growth factors and cytokines, such as TGF‐β1, activate Sp1, which causes the overexpression of VEGF. Furthermore, in cultured endothelial cells, HIF‐1α was rapidly increased and then decreased during acute hypoxia, whereas the expression of HIF‐2α began later during prolonged hypoxia.^16^ In the present study, the overexpression of VEGF in EMCS cells may result from the unknown underlying mechanisms, including the activation of Sp1 and HIF‐2α transcriptional factors.

The third limitation of the present study is that we could not compare the immunological profile of pleural metastatic lesions with that of the primary tumor, because the primary tumor was archived at another hospital. Previous studies commonly showed abundant M2 macrophages and sparse CD8^+^ T cells in EMCS developing in any regions.^4^, ^5^ However, several studies of other malignancies and preclinical models showed the difference in the tumor immune microenvironment between primary tumor and metastatic lesions.^17^, ^18^ We cannot deny the possibility of immunological alterations during the metastasis to the pleural cavity with specific circumstance.

In conclusion, EMCS cells with low tumor mutational burden secreted TGF‐β1 and VEGF into the tumor stroma, which induced abundant M2 macrophages, hypervascularity, and sparse CD8^+^ T cells. These findings suggest that extraskeletal myxoid chondrosarcoma is a poorly immunogenic “cold” tumor, and novel combined immunotherapy is required, such as TGF‐β1 and VEGF blockade, and specific therapy for enhancing tumor antigens.

## CONFLICT OF INTEREST

No authors report any conflicts of interest. Written, informed consent for the publication of this case report was obtained from the patient.
